# Aseptic meningitis and meningoencephalitis associated with pediatric histiocytic necrotizing lymphadenitis: a 26-case series

**DOI:** 10.3389/fimmu.2026.1893986

**Published:** 2026-08-19

**Authors:** Fang Guo, Lei Kang, Bo Li, Yanhong Jia, Xiaoyuan Wu

**Affiliations:** 1 Department of Infections Disease, Hebei Provincial Clinical Research Center for Child Health and Disease, Shijiazhuang, Hebei, China; 2 Department of Infectious Disease, Hebei Children’s Hospital, Shijiazhuang, Hebei, China; 3 Department of Pediatric Intensive Care Unit, Hebei Children’s Hospital, Shijiazhuang, Hebei, China; 4 Hebei Provincial Key Laboratory of Pediatric Cardiovascular Disease, Shijiazhuang, China

**Keywords:** aseptic meningitis, children, histiocytic necrotizing lymphadenitis, Kikuchi-Fujimoto disease, lymphadenopathy, meningoencephalitis

## Abstract

**Background and purpose:**

Aseptic meningitis (AM) and meningoencephalitis (ME) is a rare and under recognized complication of histiocytic necrotizing lymphadenitis (HNL) in children, with no large-scale series reported. The present study aims to characterize the clinical features of HNL-AM/ME and improve recognition of this condition.

**Methods:**

We retrospectively analyzed the clinical data of children with HNL-AM/ME admitted from January 2018 to January 2026, summarizing their clinical manifestations, laboratory findings, brain magnetic resonance imaging (MRI) findings,and treatment protocols.

**Results:**

Among 797 children with HNL, 26 met criteria (23 AM, 3 ME), with a male-to-female ratio of 4.2:1. Median age at onset for all cases was 10.0 years (IQR, 6.8–12.0 years), but all ME cases were ≤6 years. All 26 presented with fever and lymphadenopathy, with a median fever duration of 26.5 days (IQR, 18.0–35.3 days), and only 4 (15.4%) cases defervesced before glucocorticoids, while neurological symptoms were the initial manifestation in 11 (42.3%) cases. Leukopenia was observed in 20 (76.9%) cases. Elevated serum ferritin (>500 μg/L) was found in 5 (19.2%) cases. All 26 patients had cerebrospinal fluid (CSF) pleocytosis (median 57.0/μL; range 16–529/μL). Elevated CSF pressure (220–280 mmH_2_O) was noted in 10 children, and CSF protein was increased in 12 children. Nine (30.8%) cases exhibited linear hyperintense signals along segments of the cerebral sulci on fluid-attenuated inversion recovery (FLAIR) sequences. Only 5 patients underwent gadolinium-enhanced brain MRI, with 3 showing leptomeningeal enhancement (LME). A total of 24 cases (92.3%) received glucocorticoid treatment, 7 cases (26.9%) were combined with IVIG, and 3 ME children received immune enhancement treatment. Recurrence occurred in 8.3% (2/24) of glucocorticoid-treated patients, whereas both untreated patients relapsed. Of the 3 ME children, 2 developed epilepsy, and 1 concurrently developed macrophage activation syndrome (MAS).

**Conclusion:**

HNL-AM/ME predominantly affects school-aged boys. Typical features include recurrent fever, headache, leukopenia, CSF pleocytosis, linear sulcal FLAIR hyperintensity, LME, poor response to anti-infective therapy, and favorable response to glucocorticoids. Younger children (<6 years) with markedly elevated ferritin, and brain parenchymal involvement, may have a higher risk of severe disease. Early diagnosis and glucocorticoid therapy improve prognosis.

## Introduction

Histiocytic necrotizing lymphadenitis (HNL), also known as Kikuchi-Fujimoto disease (KFD), is a rare, self-limited lymphadenitis of unknown etiology, initially reported in Japan in 1972 (1). HNL can affect individuals of all age groups, with a higher prevalence in school-aged children. Patients often present with persistent fever, unilateral cervical lymphadenopathy, and some may be accompanied by various extranodal manifestations such as rash, hepatosplenomegaly, and arthritis (2). In rare cases, neurologic complications including aseptic meningitis (AM), meningoencephalitis (ME), subdural effusion, and cerebellar ataxia have been described (3, 4). However, the clinical features of HNL-AM/ME are non-specific and significantly overlap with those of infectious meningitis and autoimmune encephalitis. In some cases, affected children may initially present with neurological symptoms such as headache, vomiting, and seizures, followed by lymphadenopathy a couple of days or weeks later (5). This atypical presentation poses challenges in early diagnosis and can lead to misdiagnosis or underdiagnosis, hampering appropriate treatment of the disease.

Currently, existing studies on HNL-AM/ME are limited by small sample sizes, which predominantly consist of case reports. In addition, there is a lack of comprehensive series covering clinical manifestations, laboratory features, imaging characteristics, and long-term follow-up data on prognosis. Given this, we conducted a retrospective analysis of clinical data from 26 children with HNL-AM/ME who were admitted to Hebei Children’s Hospital during an 8-year period. The aim is to summarize the full clinical spectrum of HNL-AM/ME in children, enhance clinicians’ understanding of this rare disease, and facilitate early diagnosis and appropriate treatment.

## Patients and methods

### Participants

This was a retrospective, single-center cohort study. We analyzed the clinical records of children with HNL-AM/ME who were diagnosed at Hebei Children’s Hospital from January 1, 2018, to January 1, 2026. The inclusion criteria were as follows: ① age < 18 years; ② presence of HNL confirmed by cervical lymph node biopsy; ③ meeting the diagnostic criteria for AM/ME (6), including: (I) clinical symptoms or signs of central nervous system involvement (e.g., headache, vomiting, seizures, meningeal irritation, focal neurological deficits, altered consciousness, or lethargy); (II) cerebrospinal fluid (CSF) examination showing increased white blood cell (WBC) counts (>10/μL, predominantly mononuclear cells), increased protein levels (>0.4 g/L), and normal or mildly reduced glucose levels; (III) negative results from smears, cultures, and PCR tests for bacteria, fungi, Mycobacterium tuberculosis, and other common pathogens in CSF; (IV) brain MRI (plain/enhanced) showing meningeal or cerebral parenchymal abnormalities, or normal findings. The exclusion criteria were: ① congenital immunodeficiency or genetic metabolic diseases; ② meningitis or meningoencephalitis caused by rheumatic/immunological diseases, hematologic malignancies, poisoning, trauma, or other etiologies.

### Clinical data collection

The following variables were included in the clinical data: sex, age, length of hospitalization, clinical features, initial symptoms, laboratory parameters, brain MRI findings, management, and outcomes. All brain MRI scans were acquired using a 3-tesla (3T) magnetic field strength. After discharge, children were followed up via clinic visits every 2–4 weeks.

### Pathological examination of lymph nodes

Lymph node biopsy tissue was fixed in 10% neutral-buffered formalin, embedded in paraffin, and serially sectioned at 4μm before being stained with the standard hematoxylin and eosin method. This was followed by immunohistochemical staining using antibodies against CD3, CD20, CD5, CD4, CD8, CD10, CD68, CD123, and Ki-67. *In situ* hybridization for Epstein–Barr virus-encoded RNA (EBER) was also performed. The pathological findings were consistent with the typical features of HNL: the lymph node structure was partially or completely destroyed, with patchy or focal coagulative necrosis and abundant nuclear debris within the necrotic areas. Surrounding the necrotic areas, there was infiltration of histiocytes, plasmacytoid dendritic cells, and lymphocytes, whereas no neutrophils or granulomas were observed. Immunohistochemistry showed predominantly CD68+ histiocytes and CD3+ T cells.

### Ethical approval and informed consent

This study was approved by the Medical Ethics Committee of Hebei Children’s Hospital, affiliated with Hebei Medical University (No. 2024147).Written informed consent was provided by the parents or legal guardians of pediatric patients.

## Results

### Demographics and clinical data

**Figure 1:**
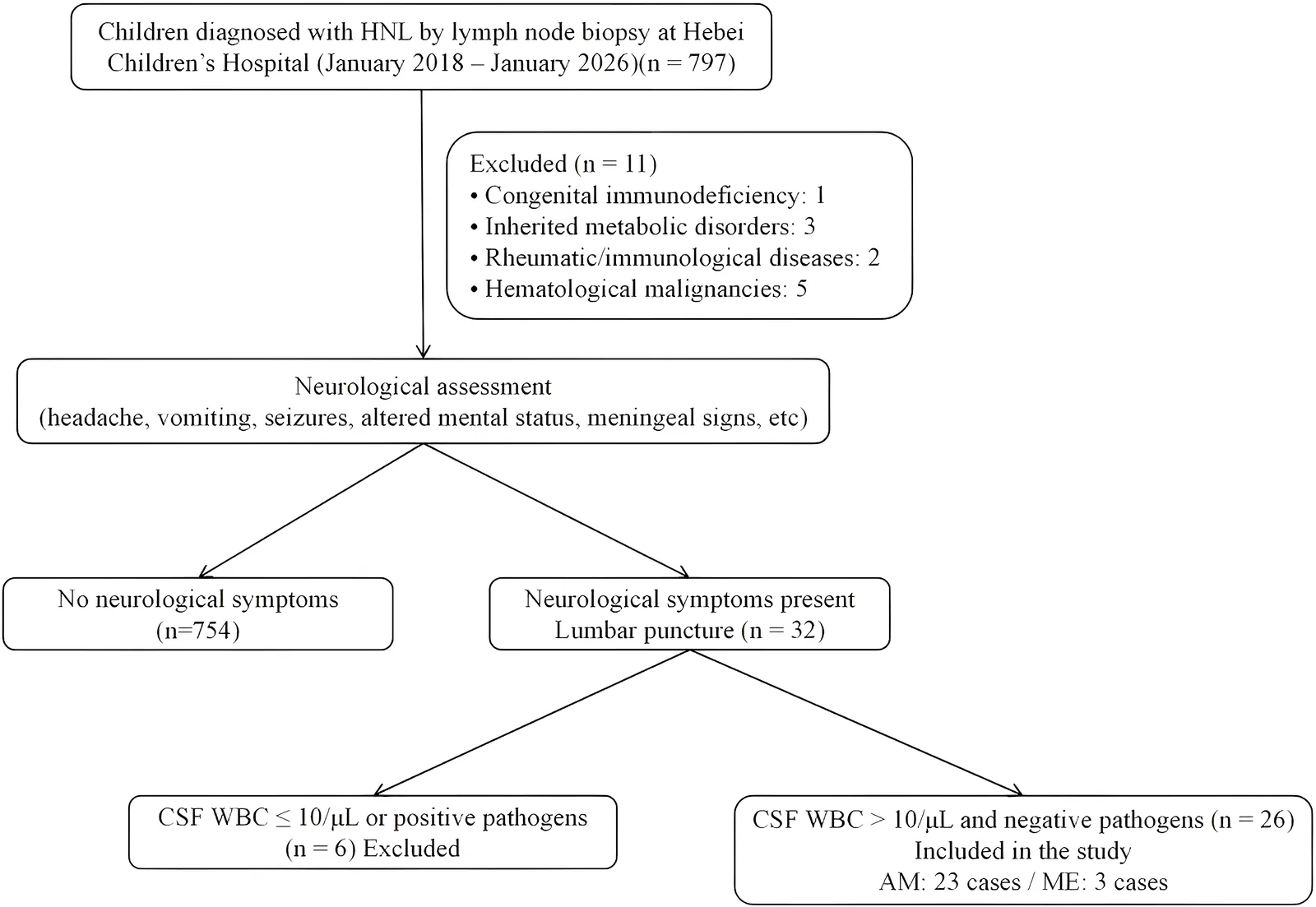
Flowchart of patient selection and enrollment.

A total of 797 children with HNL were reviewed during the study period, of whom 26 met the aforementioned inclusion criteria (**Figure 1**), yielding a detection rate of 3.3%, with 23 AM cases and 3 ME cases (**Table 1**). Among these, 21 were male (80.8%), with a male-to-female ratio of approximately 4.2:1. The median age at onset was 10.0 years (interquartile range [IQR], 6.8–12.0 years), and 6 cases (23.1%) had onset before the age of 6 years, including 3 cases with AM and 3 cases with ME. The median length of hospital stay was 20.0 days (IQR, 17.0–24.3 days). Regarding initial symptoms: 11 children presented with neurological manifestations including headache, vomiting, and seizures; 4 cases presented with fever accompanied by lymphadenopathy; and 11 cases presented with fever alone.

**Table 1:** The clinical features of patients with HNL-AM/ME.

Case no.	Age/sex	Symptoms			Laboratory data			CSF			Treatment		Outcome
		Fever	Headache	Seizures	WBC(/μL)	CRP(mg/L)	Ferritin >500(μg/L)	Cell count(/μL)	CSF/serum glucose ratio <0.5	Protein(mg/dL)	Corticosteroids	IVIG	
ME													
1	4/M	+	+	+	3.4	16	1798	26	–	0.48	+	+	S &R
2	4/M	+	+	+	0.9	207	14718	30	–	0.76	+	+	S
3	6/M	+	+	+	3.4	41	799	35	–	0.65	+	+	S
AM													
4	12/M	+	+	–	5.0	26	–	109	–	0.8	+	–	
5	12/M	+	–	–	2.7	2	–	22	–	0.15	+	–	
6	8/M	+	+	–	4.1	8	–	293	–	0.96	+	–	
7	10/M	+	+	+	3.6	22	–	160	–	0.42	+	–	
8	15/M	+	+	–	2.1	4	–	119	–	0.15	+	–	
9	8/M	+	–	–	2.4	2	–	73	–	0.11	+	–	
10	8/M	+	+	+	3.3	24	1381	224	–	1.66	+	–	
11	11/M	+	–	–	2.1	19	–	61	–	0.32	+	–	
12	12/M	+	+	–	6.7	24	–	529	–	1.91	+	+	
13	15/F	+	+	–	1.5	26	–	145	–	2.45	+	–	
14	7/M	+	–	–	1.8	12	–	46	–	0.32	+	+	
15	11/M	+	+	–	7.2	2	–	201	–	0.48	–	–	R
16	10/M	+	–	–	3.6	18.29	–	53	–	0.38	+	–	
17	3/M	+	+	+	2.1	2	–	31	–	1.15	+	+	
18	11/F	+	–	–	3.0	33	–	24	–	0.19	+	–	
19	7/M	+	+	–	3.1	11.74	–	53	–	0.36	+	–	R
20	3/M	+	+	–	4.2	8	–	26	–	0.23	–	–	R
21	12/F	+	+	–	2.0	0.5	–	310	–	0.74	+	–	
22	8/F	+	+	–	4.2	18.86	714	38	–	0.36	+	+	
23	5/F	+	–	–	3.59	4.48	–	17	–	0.3	+	–	
24	13/M	+	–	–	2.9	5.6	–	89	–	0.36	+	–	
25	11/M	+	+	–	3.8	13.76	–	134	–	0.31	+	–	
26	12/M	+	–	–	2.24	1.72	–	16	–	0.19	+	–	

–, absent; +, present.

CSF, cerebrospinal fluid; F, female; M, male; WBC, white blood cell; R, relapse; S, sequelae.

All 26 children had fever and lymphadenopathy, with a duration ranging from 7 to 74 days (median, 26.5 days; IQR, 18.0–35.3 days), and only 4 cases (15.4%) had fever subsided before the initiation of corticosteroid therapy. The predominant neurological symptoms were headache (17/26, 65.4%), vomiting (15/26,57.7%), seizures (6/26, 23.1%), altered mental status (4/26, 15.4%), and status epilepticus (2/26, 7.7%). Other accompanying manifestations included rash (13/26, 50.0%), chills (10/26, 38.5%), oral ulcers (5/26, 19.2%), hepatosplenomegaly (5/26, 19.2%), and arthralgia (4/26, 15.4%). Two children with ME (2/26, 7.7%) developed respiratory failure and required mechanical ventilation, and one child with ME (1/26, 3.8%) was diagnosed with concurrent macrophage activation syndrome (MAS).

### Laboratory findings

At the time of HNL diagnosis, a reduced white blood cell (WBC) count was detected in 20 children (76.9%), of whom 10 (38.5%) had already presented with leukopenia on admission. Decreased hemoglobin (Hb) levels were observed in 7 children (26.9%). Elevated C-reactive protein (CRP) was found in 15 children (57.7%), and an elevated erythrocyte sedimentation rate (ESR) in 17 children (65.4%). Five children (19.2%) had elevated serum ferritin (>500 μg/L): two cases (8.7%) in the AM group (714μg/L and 1381 μg/L) and three cases (100%) in the ME group (799μg/L, 1798μg/L, and 14718 μg/L). The overall median inflammatory burden index (IBI) was 22.0 (IQR, 4.1-48.8), while that of the ME group was 16.9, 51,and 98.9. Elevated alanine aminotransferase (ALT) levels were measured in 12 children (54.5%) (range, 122.0–578.0U/L).

Six children (23.1%) tested positive for autoantibodies, with some having more than one type. Specifically, anti-Smith was found in two, anti-Ro52 in four, anti-SS-A in one, and anti-Jo-1 in one. Two children (7.7%) were antinuclear antibody-positive with low titers. Among the 23 children (88.5%) who underwent lymphocyte subset analysis, CD4+ T lymphocyte levels were reduced in 11 cases and CD8+ T lymphocyte levels were increased in 11 cases, with seven of the latter being male and three belonging to the ME group. Bone marrow puncture excluded hematologic malignancies in all cases.

All 26 patients had CSF pleocytosis, with a predominance of mononuclear leukocytes. The overall median white blood cell count in the CSF was 57.0/μL (IQR 29.0–148.8/μL), range 16–529/μL (reference range 0–10/μL), while the cell counts in the ME group were 26.0, 30.0, and 35.0/μL. Twelve children (46.2%) had elevated CSF protein (>400mg/L), with a median of 0.8g/L (range 0.11–2.45g/L). No child had abnormal CSF/serum glucose ratio (reference range>0.5). Ten cases showed elevated intracranial pressure (range 220-280mmH_2_O; reference range 80-180mmH_2_O). CSF bacterial cultures and polymerase chain reaction (PCR) assays for *adenovirus, cytomegalovirus (CMV)*, *Epstein–Barr virus (EBV)*, *herpes simplex virus types 1 and 2 (HSV-1, HSV-2)*, *Mycoplasma pneumoniae (MP)*, *varicella zoster virus (VZV)*, and *enterovirus* were all negative. Furthermore, metagenomic next-generation sequencing (mNGS) was performed on CSF samples from 12 patients, and no infectious pathogens were detected.

### MRI findings

**Figure 2:**
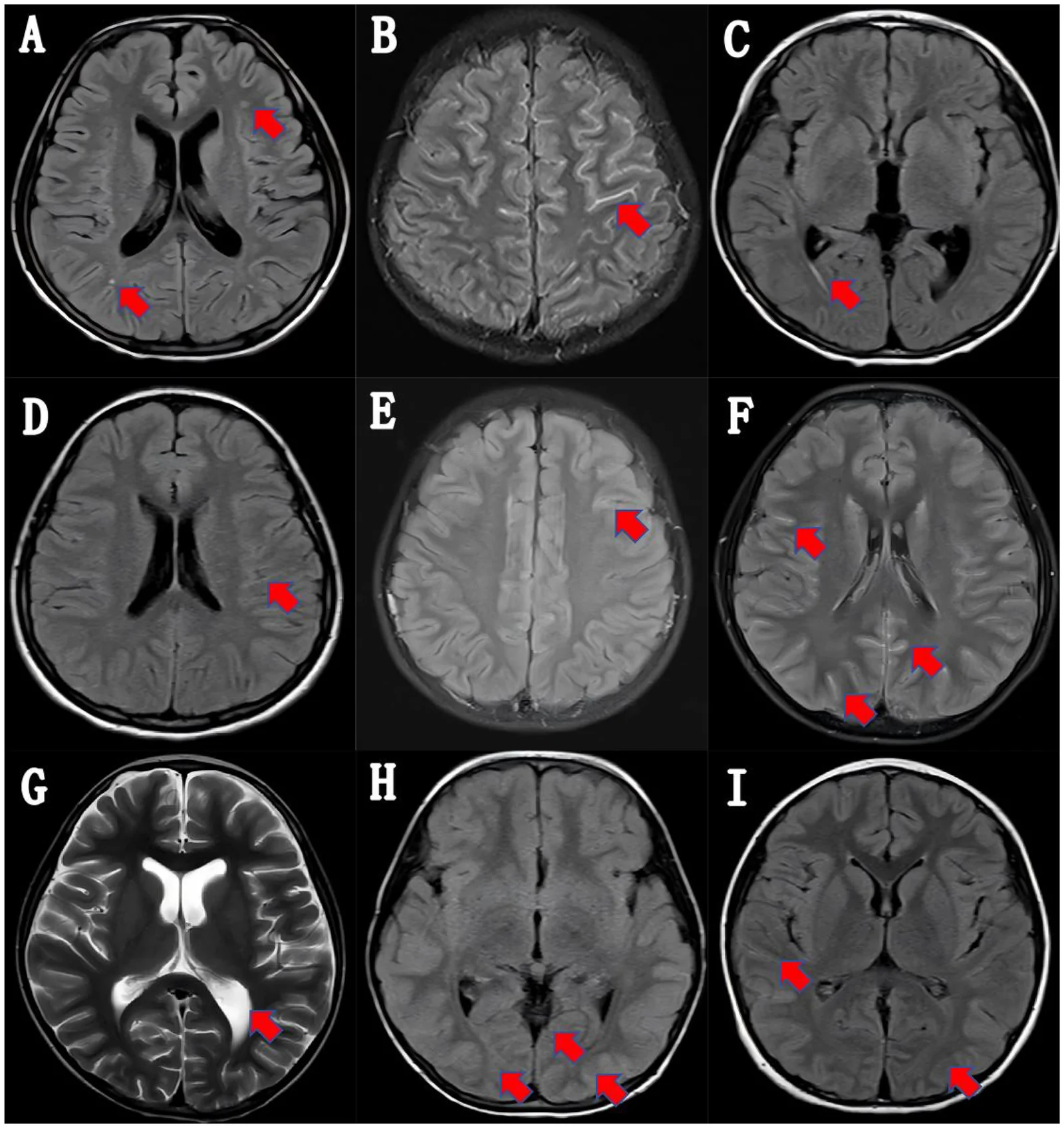
Brain magnetic resonance imaging (MRI) of patients with HNL-AM/ME. **(A)** Spotty or patchy distribution of abnormal signals in the frontal and occipital lobes; **(B)** Leptomeningeal enhancement sign; **(C)** Enlarged bilateral lateral ventricles, and hyperintensity in the right trigone on FLAIR; **(D)** Hyperintensity in the left frontoparietal sulci on FLAIR; **(E)** Widened cerebral sulci and mild focal LME; **(F)** Multifocal intracranial LME; **(G)** Widened cerebral sulci and mildly widened left occipital horn; **(H)** Gyral swelling of left parieto-occipital lobe, abnormal signals in left occipitoparietal and bilateral cingulate gyri, with linear hyperintense signals of sulci on FLAIR; **(I)** Widened cerebral sulci and hyperintensity in the right tempora sulci on FLAIR.

All 26 children underwent brain MRI. Among these patients, 9 (30.8%) exhibited linear hyperintense signals along segments of the cerebral sulci on fluid-attenuated inversion recovery (FLAIR) sequences (**Figure 2**), while 7 (26.9%) showed no abnormal signals. Brain MRI revealed multiple punctate or patchy abnormal signals in the white matter of the bilateral frontal, parietal, and occipital lobes in 6 patients, suggesting focal dysmyelination or demyelinating changes. Four cases (15.4%) presented with widened cerebral sulci and extracerebral spaces. Only 5 patients underwent gadolinium-enhanced brain MRI, with 3 showing leptomeningeal enhancement (LME) (**Figure 2**), including 1 case (1/23, 4.3%) in the AM group and 2 cases (2/3, 66.7%) in the ME group.

### Ultrasound and pathological examination of lymph node

All 26 pediatric patients underwent ultrasound examination of the lymph nodes. Findings included cortical thickening with loss of the echogenic hilum, as well as reduced, linearized, or absent medullary echogenicity. The mean longest diameter of the lymph nodes was 21.7 ± 4.2 mm, and the mean ratio of the longest to the shortest diameter was 2.5 ± 0.5. Pathological examination of the 26 lymph node specimens revealed destruction of the nodal architecture, with only a small amount of residual lymphoid tissue and follicles at the periphery. Extensive necrosis and karyorrhectic debris, along with histiocytic hyperplasia, were observed. Immunohistochemical staining showed positivity for CD3 (T cells), CD20, CD21 (dendritic cells), and CD68. *In situ* hybridization for Epstein-Barr virus-encoded RNA (EBER) was negative.

### Treatment and outcomes

All 26 children received empiric anti-infective therapy due to concerns about intracranial infection. Among them, 22 (84.6%) were given empiric intravenous acyclovir for suspected herpes simplex virus (HSV) encephalitis, and 22 cases (84.6%) received antibiotics for 1–2 weeks, including third-generation cephalosporins (n=16), azithromycin (n=7), vancomycin (n=2), and meropenem (n=1). Combination antibiotic therapy was administered to 9 patients (34.6%). However, no patient showed a favorable response to anti-infective treatment. All anti-infective agents were discontinued once a definitive diagnosis was established by lymph node biopsy.

Following confirmation of HNL-AM/ME, 24 patients (92.3%) received glucocorticoid therapy. The median interval from symptom onset to glucocorticoid initiation was 26.5 days (IQR: 18.3-37.8 days). Two patients (7.7%) did not receive glucocorticoids, as their fever and neurological symptoms resolved with nonsteroidal anti-inflammatory drugs (NSAIDs). The standard glucocorticoid regimen consisted of intravenous methylprednisolone sodium succinate (IVMP) at 1–2 mg/(kg·d) for 1 week, followed by oral prednisone tapered over 6–8 weeks. Seven of the 24 children (29.2%) also received intravenous immunoglobulin (IVIG) at 1 g/(kg·d) for 2 days. Three patients with ME underwent intensified immunotherapy combining high-dose IVMP with IVIG. The high-dose IVMP regimen was 10 mg/(kg·d) for 3 days, followed by 5 mg/(kg·d) for 3 days, then 2.5 mg/(kg·d) for 3 days, and subsequently tapered to the standard glucocorticoid dose.

After discharge, all 26 patients were followed up at the outpatient clinic for a mean of 12.6±7.5 months (range, 3-24 months). At each visit, we performed a standardized neurological examination, assessing both symptoms (headache, vomiting, and seizures) and signs (mental status, nuchal rigidity, muscle strength, reflexes, and coordination). Among the 24 children who received glucocorticoids, only two relapsed, whereas both of the two children who did not receive glucocorticoids relapsed. Among the four relapsed children, one presented again with HNL-AM after 1 month, while the other three were not accompanied by neurological manifestations. One child relapsed twice, with intervals of 4 years and 6 years from the first onset, respectively. Two children developed status epilepticus during hospitalization, were subsequently diagnosed with epilepsy, and received regular antiepileptic therapy. To date, none of the patients, including six children with positive autoantibodies, have progressed to autoimmune rheumatic diseases.

## Discussion

HNL is a benign, self-limiting disease of unknown etiology. It has been reported worldwide but is more common in Asian populations, especially in Japan, South Korea, and China (7). Epidemiological data show that HNL is more prevalent in females. However, in children, the proportion of boys under 10 years of age is relatively high, and the female advantage gradually becomes more prominent after puberty (8). The disease is characterized by fever and superficial lymphadenopathy, and the course usually lasts from several weeks to several months before spontaneous resolution. Some children may progress to autoimmune rheumatic diseases, such as systemic lupus erythematosus and Sjögren’s syndrome, or may be complicated by macrophage activation syndrome, liver and kidney failure, and central nervous system damage (9, 10). The neurological manifestations of HNL are complex and diverse. However, there is a lack of systematic studies on the incidence, clinical features, treatment strategies, and long-term prognosis of aseptic HNL-AM/ME in children.

In this study, AM and ME accounted for 26 of 797 (3.3%) children diagnosed with HNL during the same period, with a median age at onset of 10 years, consistent with the age distribution of pediatric HNL. However, the male-to-female ratio was 4.2:1 (80.8% male), suggesting a more pronounced male predominance compared with the general pediatric HNL population (11). Several factors may explain this gender difference. First, HNL is closely associated with T-cell-mediated immune responses, and the infiltrating cells in lesional tissues are predominantly CD8+ T lymphocytes (12). Male individuals tend to exhibit a more pronounced cytotoxic T-cell response, whereas females show stronger humoral immunity and immunoregulatory capacity (13). Second, boys with inflammatory diseases have significantly higher levels of pro-inflammatory cytokines (IFN-γ, IL-6, TNF-α) than girls (14). In the present study, 11 children with HNL-AM/ME had elevated peripheral blood CD8+ T lymphocytes, and 63.6% (7/11) of these were boys, suggesting a potential role of cytotoxic immune activation in the male predominance of HNL-AM/ME. Furthermore, estrogen protects the blood-brain barrier from inflammatory damage (15). Thus, before puberty, boys have low estrogen levels and consequently higher blood-brain barrier permeability. Animal studies further confirm that under inflammatory or injurious conditions, male mice suffer more severe blood-brain barrier damage than female mice, suggesting that estrogen deficiency may exacerbate barrier disruption (16). Multicenter prospective studies are needed to corroborate these findings.

This study revealed that the manifestations of HNL-AM/ME were complex and diverse: ①Fever was persistent and refractory, with a median duration of 26.5 days. There was no favorable response to anti-infective therapy, and only 15.4% of patients achieved defervescence before corticosteroid administration. ②Neurological symptoms were the initial presentation in 42.3% of children, with headache as the most common symptom, followed by vomiting and seizures. Typical lymphadenopathy was delayed by several days to weeks, which consistent with previous studies (3). The underlying mechanism may involve increased permeability of cerebral capillary endothelial cells, leading to vasogenic cerebral edema. Furthermore, inflammation-induced disruption and adhesion of the meningeal surface may impair CSF absorption and circulation, subsequently leading to elevated intracranial pressure (17). ③Over three quarters of the patients (n=20) exhibited decreased peripheral blood leukocyte counts, a pattern that differs from bacterial meningitis (where leukocyte counts are typically elevated) and viral meningitis (where leukocyte counts are normal or mildly increased). ④In the present study, additional findings included CSF pleocytosis, elevated CSF protein levels, elevated intracranial pressure, FLAIR hyperintense signals along the cerebral sulci or leptomeningeal enhancement on brain MRI. These findings were difficult to distinguish from viral encephalitis and tuberculous meningitis, consistent with previous reports (18). These findings are consistent with the report by Liu (5), who also identified headache, seizures, and elevated CRP as significant indicators of CNS involvement in pediatric HNL. Therefore in children presenting with prolonged fever, encephalitis or meningitis, decreased peripheral blood leukocyte counts, and no response to anti-infective therapy, clinicians should maintain a high index of suspicion for HNL-AM/ME. Early superficial lymph node ultrasound should be performed to guide biopsy decisions based on lymph node morphology and to help differentiate from malignant diseases such as lymphoma.

Most children with HNL follow a benign, self-limiting course. Approximately half experience spontaneous resolution after lymph node biopsy (2) or rapid defervescence following short-term NSAID therapy. Accordingly, NSAIDs are widely recommended as first-line treatment. Corticosteroids, as immunomodulatory agents, are reserved for persistent fever despite NSAID use, recurrent episodes, or severe complications (MAS or neurological involvement) (19). In a study of 19 children with HNL-related central nervous system disease, Huang (20) reported a shorter clinical course in treated patients. In the present study, 22 children presented with persistent high fever refractory to NSAIDs. Of these, 15 cases became afebrile after receiving standard glucocorticoid regimen, while the remaining 7 cases responded poorly to IVMP alone but achieved defervescence with the addition of IVIG; three of these required IVMP pulse therapy. An additional 4 children defervesced before glucocorticoid initiation, two of whom received oral prednisone post-diagnosis. Among glucocorticoid-treated children, two relapsed with fever and lymphadenopathy. Recurrent aseptic meningitis associated with HNL is rare. In this study, among two HNL-AM children who did not receive glucocorticoids, one relapsed after one month. Komagamine (21) described a case with seven recurrences over seven years, in which NSAIDs provided only transient control (shortest inter-recurrence interval: 11 days), whereas the other three patients with recurrent meningitis did not relapse after glucocorticoid therapy. Our relapse rates are consistent with the favorable outcomes reported by Ahmed (3), who noted that corticosteroids were effective in most HNL patients with neurological involvement. Therefore, for children with HNL-AM/ME, once contraindications are excluded, early glucocorticoid therapy may be considered during the acute phase, with additional IVIG and/or methylprednisolone pulse therapy based on disease severity.

Among the 26 children, two (7.7%) required mechanical ventilation due to status epilepticus and concurrent central respiratory failure, and one (3.8%) developed MAS. All three children were ≤6 years of age, exhibited brain parenchymal involvement, had markedly elevated ferritin and IBI, and showed mildly elevated CSF white blood cell counts. They did not respond to standard glucocorticoid regimen but improved after receiving intensified immunotherapy (combining high-dose IVMP with IVIG), fitting a pattern characterized as “young age and high inflammation”. Possible mechanisms underlying this severe phenotype include the following: ① In young children, the blood–brain barrier (BBB) is not fully developed, with incomplete tight junctions and increased endothelial permeability; combined with the high reactivity of microglia to IFN-γ/TNF-α (22), this renders the immature brain more vulnerable to parenchymal edema and irreversible damage under similar inflammatory stimuli. ② Pathological specimens of HNL show abundant CD68+ histiocytes and CD123+ plasmacytoid dendritic cells aggregated around necrotic areas, and these activated immune cells are a major source of ferritin (23). Elevated ferritin in CNS inflammation can worsen oxidative stress and inflammatory injury via the NF-κB/p65- mediated oxidative stress pathway (24). ③ A high overall inflammatory burden, coupled with immune cell imbalance, may synergistically disrupt BBB integrity and facilitate the migration of peripheral inflammatory factors and immune cells into the CNS. These observations suggest the following: in children with HNL presenting with neurological manifestations, when both “young age” and the “high inflammation” phenotype are present—even in the absence of a marked CSF pleocytosis—clinicians should maintain a high index of suspicion for severe meningoencephalitis, initiate intensified immunosuppressive therapy as early as possible, and dynamically monitor ferritin and IBI levels to guide prognostication.

This study had several limitations. First, the single-center, retrospective design introduces potential selection bias. Moreover, the small sample size of the ME group limits the statistical power of subgroup analyses. Second, given the extended study period, CSF cytokine profiles were not uniformly measured, precluding an in-depth investigation of the immune mechanisms underlying HNL-AM/ME. Third, enhanced cranial MRI was not performed in all children, which may have led to an underestimation of the true frequency of meningeal enhancement. Finally, treatment was clinician-assigned rather than randomized; therefore, causal inferences should be drawn with caution. Future multicenter prospective cohort studies, incorporating CSF immunological profiling and long-term neurodevelopmental follow-up, are needed to further elucidate the pathogenesis of HNL-AM/ME and to optimize therapeutic strategies.

## Conclusion

HNL-AM/ME is more common in school-aged boys. Clinically, it is characterized by persistent fever, leukopenia, CSF pleocytosis, linear sulcal FLAIR hyperintensity, and LME. In our cohort, anti- infective therapy was generally ineffective, whereas corticosteroid administration appeared to be beneficial. We observed that younger age (under 6 years), markedly elevated ferritin, and brain parenchymal involvement appeared to be associated with more severe disease, whereas CSF pleocytosis showed no clear correlation with severity. However, given the retrospective design and the lack of a control group, these exploratory findings should be interpreted with caution and will require further validation in prospective studies.

## Data Availability

The datasets presented in this study can be found in online repositories. The names of the repository/repositories and accession number(s) can be found in the article/supplementary material.

## References

[1] Kikuchi M . Lymphadenitis showing focal reticular cell hyperplasia with nuclear debris and phagocytes. Acta Haematologica Japonica. (1972) 35:379–80.

[2] Choi S Choi HS Ryu YJ Kim JY Paik JH Ahn S . Characterization of Kikuchi-Fujimoto disease in children and risk factors associated with its course. J Pediatr. (2023) 260:113515. doi: 10.1016/j.jpeds.2023.113515 37244579

[3] Ahmed HS D'Souza L M S V Sache MS . Neurological manifestations and complications of Kikuchi-Fujimoto disease: a comprehensive systematic review. Clin Neurol Neurosurg. (2025) 251:108818. doi: 10.1016/j.clineuro.2025.108818 40056750

[4] Moon JS Il Kim G Koo YH Kim HS Kim WC Kim OJ . Kinetic tremor and cerebellar ataxia as initial manifestations of Kikuchi-Fujimoto's disease. J Neurol Sci. (2009) 277:181–3. doi: 10.1016/j.jns.2008.10.021 19027125

[5] Liu B Sun Y Hu B Shi WY Chen TM Liu LL . Kikuchi-Fujimoto disease concurrent with aseptic meningitis or encephalitis in children: a case-control study. BMC Pediatr. (2025) 25:287. doi: 10.1186/s12887-025-05648-y 40221671 PMC11992700

[6] Etemadifar M Fereidan- Esfahani M Sedaghat N Kargaran PK Mansouri AR Abhari AP . Non-infectious meningitis and CNS demyelinating diseases: A conceptual review. Rev Neurol (Paris) (2023) 179(6):533–47. 10.1016/j.neurol.2022.10.006 36781321

[7] Mahajan VK Sharma V Sharma N Rani R . Kikuchi-Fujimoto disease: A comprehensive review. World J Clin Cases. (2023) 11(16):3664–79. doi: 10.12998/wjcc.v11.i16.3664 PMC10294163 37383134

[8] Kim HY Jo HY Kim SH . Clinical and laboratory characteristics of Kikuchi-Fujimoto disease according to age. Front Pediatr. (2021) 9:745506. doi: 10.3389/fped.2021.745506 34796153 PMC8593182

[9] Zhang D Su GX Wu FQ Zhu J Kang M Xu YJ . Clinical features and prognosis of 118 children with histiocytic necrotizing lymphadenitis. Zhonghua Er Ke Za Zhi. (2023) 61:533–7. doi: 10.3760/cma.j.cn112140-20230110-00020 37312465

[10] Liu C Jin Y Huang H Ding F Yang Z Xu X . Kikuchi-Fujimoto disease as the initial manifestation of systemic lupus erythematosus complicated with macrophage activation syndrome: two case reports and a review of literature. BMC Pediatr. (2022) 22:673. doi: 10.1186/s12887-022-03703-6 36414954 PMC9682638

[11] Selvanathan SN Suhumaran S Sahu VK Chong CY Tan NWH Thoon KC . Kikuchi-Fujimoto disease in children. J Paediatr Child Health. (2020) 56:389–93. doi: 10.1111/jpc.14628 31576642

[12] Yamashita T Momose S Imada H Takayanagi N Murakami C Nagata M . The significance of T-BET-positive CD8 T-cells with diminished CD5 expression in Kikuchi-Fujimoto disease. J Clin Exp Hematop. (2024) 64:183–90. doi: 10.3960/jslrt.24019 39085130 PMC11528254

[13] Mitul MT Kastenschmidt JM Sureshchandra S Wagoner ZW Sorn AM Mcllwain DR . Tissue-specific sex differences in pediatric and adult immune cell composition and function. Front Immunol. (2024) 15:1373537. doi: 10.3389/fimmu.2024.1373537 38812520 PMC11133680

[14] Rajamanickam A Kumar NP Venkataraman A Varadarjan P Selladurai E Sankaralingam T . Sex-specific differences in systemic immune responses in MIS-C children. Sci Rep. (2024) 14:1720. doi: 10.1038/s41598-024-52116-1 38243064 PMC10799056

[15] Kurmann L Azzarito G Leeners B Rosselli M Dubey RK . 17β-estradiol abrogates TNF-α-induced human brain vascular pericyte migration by downregulating miR-638 via ER-β. Int J Mol Sci. (2024) 25:11416. doi: 10.3390/ijms252111416 39518968 PMC11547073

[16] Velmurugan GV Prajapati S Tran S Miller C Burke B Hubbard WB . Sex-dependent blood-brain barrier alterations following repeated mild blast traumatic brain injury at varying inter-injury intervals. Exp Neurol. (2025) 392:115325. doi: 10.1016/j.expneurol.2025.115325 40451452 PMC12302826

[17] Tian M Li M Zhang C Luo Q Wang A Li D . The neurobiological regulatory mechanism of brain edema. Front Cell Neurosci. (2026) 20:1777039. doi: 10.3389/fncel.2026.1777039 41988389 PMC13076164

[18] Tseng TY Lin YJ . Aseptic meningitis with cerebellitis secondary to histiocytic necrotizing lymphadenitis (Kikuchi-Fujimoto disease): a case report. Acta Neurol Taiwan. (2025) 34:106–8. doi: 10.4103/ANT.ANT_113_0007 40434841

[19] The Subspecialty Group of Immunology, the Society of Pediatrics, Chinese Medical Association . Consensus on the diagnosis and management of histiocytic necrotizing lympadenitis in children. Chin J Evid Based Pediatr. (2025) 20:161–6. doi: 10.3969/j.issn.1673-5501

[20] Huang Y Wang X Wang L Wang J Tan C Cheng M . Kikuchi-Fujimoto disease associated with the central nervous system in children. Pediatr Discov. (2023) 1:e23. doi: 10.1002/pdi3.23 40625714 PMC12118243

[21] Komagamine T Nagashima T Kojima M Kokubun N Nakamura T Hashimoto K . Recurrent aseptic meningitis in association with Kikuchi-Fujimoto disease: case report and literature review. BMC Neurol. (2012) 12:112. doi: 10.1186/1471-2377-12-112 23020225 PMC3570427

[22] Di Martino E Rayasam A Vexler ZS . Brain maturation as a fundamental factor in immune-neurovascular interactions in stroke. Transl Stroke Res. (2024) 15:69–86. doi: 10.1007/s12975-022-01111-7 36705821 PMC10796425

[23] Dwivedi M Singh S Tripathi AK Singh M Rani D . Cytological features of Kikuchi-Fujimoto disease: a multicenter study of 30 cases. Cureus. (2025) 17:e83462. doi: 10.7759/cureus.83462 40462807 PMC12133023

[24] Singh G Singh A Mishra S Singh D Kumar A . Intracellular iron accumulation induces inflammatory and oxidative status of the host after Japanese encephalitis viral infection. Mol Neurobiol. (2024) 61:175–87. doi: 10.1007/s12035-023-03538-x 37594653

